# A survey of *Onchocerca fasciata* infection in camels (*Camelus bactrianus*) with notes on morphology

**DOI:** 10.1371/journal.pone.0214477

**Published:** 2019-04-04

**Authors:** Zhi-Chao Yu, Wei Zhang, Bin Li, Xiao-Ping Luo, Rui Wang, Xiao-Ye Yang

**Affiliations:** 1 College of Veterinary Medicine, Inner Mongolia Agricultural University, Hohhot, Inner Mongolia, China; 2 College of Veterinary Medicine, Xinjiang Agricultural University, Urumchi, Sinkiang, China; Federal University of Agriculture, Abeokuta, NIGERIA

## Abstract

The goal of this study was to provide insight into the pathogenicity of *Onchocerca fasciata* in *Camelus bactrianus* to help control onchocerciasis. From November 2015 to January 2016, the prevalence and severity of onchocerciasis were recorded in 152 camels. Nodules containing *Onchocerca* were collected and observed. Adult parasites were extracted from the nodules and identified via light microscopy as well as by partial sequencing of the cytochrome oxidase subunit I (COI) gene. The sequences were examined and compared to similar sequences from other *Onchocerca* species. In total, 80.3% of camels were parasitized. The severity of infection varied, as camels harboured between one and fifteen nodules. The morphology and the cuticle differed in both sexes and displayed considerable variation in the thickness and structure of different body parts. Identification was further confirmed using molecular biology methods. This study provides a comprehensive morphological description of *Onchocerca fasciata* isolated from camels. The prevalence and intensity of infection (assessed via nodules) varied in the Bactrian camels. The structure of the cuticle was an important morphological feature for species differentiation in *Onchocerca*. Based on our data, the morphological assessment of *O*. *fasciata* represents a reliable method to characterize other *Onchocerca* species.

## Introduction

Zoonotic onchocerciasis is a disease that affects animals and humans and is caused by various species of filarial nematode in the genus *Onchocerca* [1]. Most representatives of *Onchocerca* primarily infect cattle (*O*. *armillata*, Railliet and Henry, 1909; *O*. *gibsoni*, Cleland and Johnston, 1910; *O*. *gutturosa*, Neumann, 1910; *O*. *lienalis*, Stiles, 1892; *O*. *ochengi*, Bwangamoi, 1969) [2–6], horses (*O*. *cervicalis*, Railliet and Henry, 1910) [7], wild boars (*O*. *dewittei japonica*, Uni, Bain and Takaoka, 2001) [8], deer (*O*. *cervipedis*, Wehr and Dikmans, 1935; *O*. *flexuosa*, Wedl, 1856; *O*. *jakutensis*, Guvanov, 1964) [9–11], wolves and dogs (*O*. *lupi*, Rodonaja, 1967) [12]. *O*. *volvulus* infects humans and causes a serious disease called “river blindness”. This disease is transmitted by the blackfly (*Simulium* spp.) in many parts of the world and remains a threat to millions of people [13].

Three species of Onchocerca have been reported in camels: *O*. *fasciata* (Railliet and Henry, 1910) [14], *O*. *armillata* and *O*. *gutturosa* [15,16]. Of these species, *O*. *fasciata* is the most prevalent and can be identified based on its morphological characteristics as well as via molecular techniques such as mitochondrial gene sequencing [17].

*O*. *fasciata* is a subcutaneous filarial nematode (Spirurida: Onchocercidae) for which the camel is the only host. Adult worms live in the nuchal ligaments, subcutaneous connective tissues and the undersides of the hide. However, nematodes have also been detected in other parts of the body, where well-developed fibrous tissue nodules form. Nodule formation is a result of the host immune reaction to the parasite. Adults are enclosed by fibrous tissue that is strongly infiltrated by inflammatory cells. These nodular lesions have a detrimental impact on meat and leather quality. Moreover, in heavily infected camels, the nodules may be mistaken for tuberculous lymph nodes, alarming consumers and resulting in the loss of commercial value [18,19].

Camels, which are an ideal host, comprise two species: one-humped camels (*Camelus dromedarius*) and two-humped camels (*Camelus bactrianus*). One-humped camels are distributed in Africa and are found in Sudan, Somalia, Egypt and certain Asian countries; the majority of two-humped camels live in China and Mongolia. The Bactrian population is approximately at about 2 million animals [20,21].

To improve disease prevention, it is important to carry out studies on camel’s onchocerciasis. Thus, we recorded the prevalence and severity of onchocerciasis in the local camel population to evaluate its potential impact on camel breeding in Inner Mongolia, China. Parasites were collected to identify *Onchocerca* spp. via morphology and mitochondrial cytochrome oxidase subunit I (COI) sequence analysis, and a comprehensive description of the morphological characteristics is provided.

## Materials and methods

This study was performed in the Bactrian camel breeding regions (Alashan: 39°04′N, 105°22′E; Bayannaoer: 41°04′N, 107°04′E) of Inner Mongolia. Between November 2015 and January 2016, 152 camels (117 from Alashan and 35 from Bayannaoer) were investigated after slaughter. The camels were classified into three age groups: young (≦5 years old), adult (6–12 years old) and old (≧13 years old). The nuchal ligaments, subcutaneous tissues of the neck and underside of the hide were examined. Nodules were collected, measured and classified by size as small (diameter <15 mm), medium (15–30 mm) or large (>30 mm). Nodule condition was also noted (soft or calcified), and then the samples were frozen for later study.

Several intact adult worms were collected from nodules and stored in 70% ethanol. For light microscopy, the worms were transparentized in glycerine. Body length and width, oesophagus length, and spicule lengths were measured (in millimetres).

We next sequenced the mitochondrial COI gene in the parasites. The sequences were examined by BLAST and compared to similar sequences from GenBank that corresponded to other *Onchocerca* species (Table 1). Phylogenetic analyses were performed using DNAStar 7.1. Rough phylogenetic estimations were performed using neighbour-joining (NJ) methods, and 1,000 bootstrap replicates were performed using MEGA 5.0.

**Table 1:** The mitochondrial COI gene in different species of *Onchocerca*.

Onchocerca species	Host source	Collection site	GenBank accession no.
**Onchocerca eherhardi**	Japanese deer	Japan	AM749268.1
**Onchocerca fasciata**	Camel	China	JQ316672.1
**Onchocerca flexuosa**	Red deer	USA	HQ214004.1
**Onchocerca gibsoni**	Cattle	Australia	AJ271616.1
**Onchocerca gutturosa**	Cattle	North Cameroon	AJ271617.1
**Onchocerca lupi**	Dog	Portugal	GU365877.1
**Onchocerca ochengi**	Cattle	North Cameroon	AJ271618.1
**Onchocerca skrjabini**	Japanese deer	Japan	AM749270.1
**Onchocerca suzukii**	Rupicaprinae	Japan	AM749275.1
**Onchocerca volvulus**	Human	Africa	AF015193.1

## Results

### Infection prevalence and histopathology

Subcutaneous nodules were found in 122 of 152 (80.3%) camels examined, and the severity of infection varied between one and fifteen nodules. The camels from Alashan had a higher prevalence (88.9%) of disease than those from Bayannaoer (51.4%). Nodules were primarily found in the nuchal ligaments and subcutaneous connective tissue between the third and sixth cervical vertebrae, were ovoid or flat cysts and were classified as soft or hard (calcified).

A total of 617 nodules were measured. The overall distribution frequency of nodule size in different age groups indicated that more than half of the nodules (54.5%, n = 336) were medium-sized, with lower percentages of small (24.8%, n = 153) and large nodules (20.7%, n = 128). In both young and adult camels, 56.6% (n = 84) and 58.3% (n = 213) of the nodules, respectively, were 15–30 mm in diameter. However, in old camels, 54.9% (n = 55) were over 30 mm in diameter. The overall percentages of soft nodules and calcified nodules were 35.4% and 64.6%, respectively. Soft nodules decreased with camel age, from 52.6% in young camels to 37.4% and 2.0% in adult and old camels respectively. Unlike soft nodules, the presence of calcified nodules increased with camel age, from 47.4% in young camels to 62.6% and 98.0% in adult and old camels, respectively (Table 2).

**Table 2:** Characteristics of onchocercal nodules in camels at various ages.

Age group (years)	No. of infected camels	Total no. of nodules	Nodules (%) (size (mm))			Soft nodules (%)	Calcified nodules (%)
			<15	15–30	>30		
**Young (≦5)**	27	149	35.5	56.6	7.9	52.6	47.4
**Adult (6–12)**	79	367	25.1	58.3	16.6	37.4	62.6
**Old (≧13)**	16	101	7.8	37.3	54.9	2.0	98.0
**Total**	122	617	24.8	54.5	20.7	35.4	64.6

### Morphological description of *O*. *fasciata*

**Fig 1:**
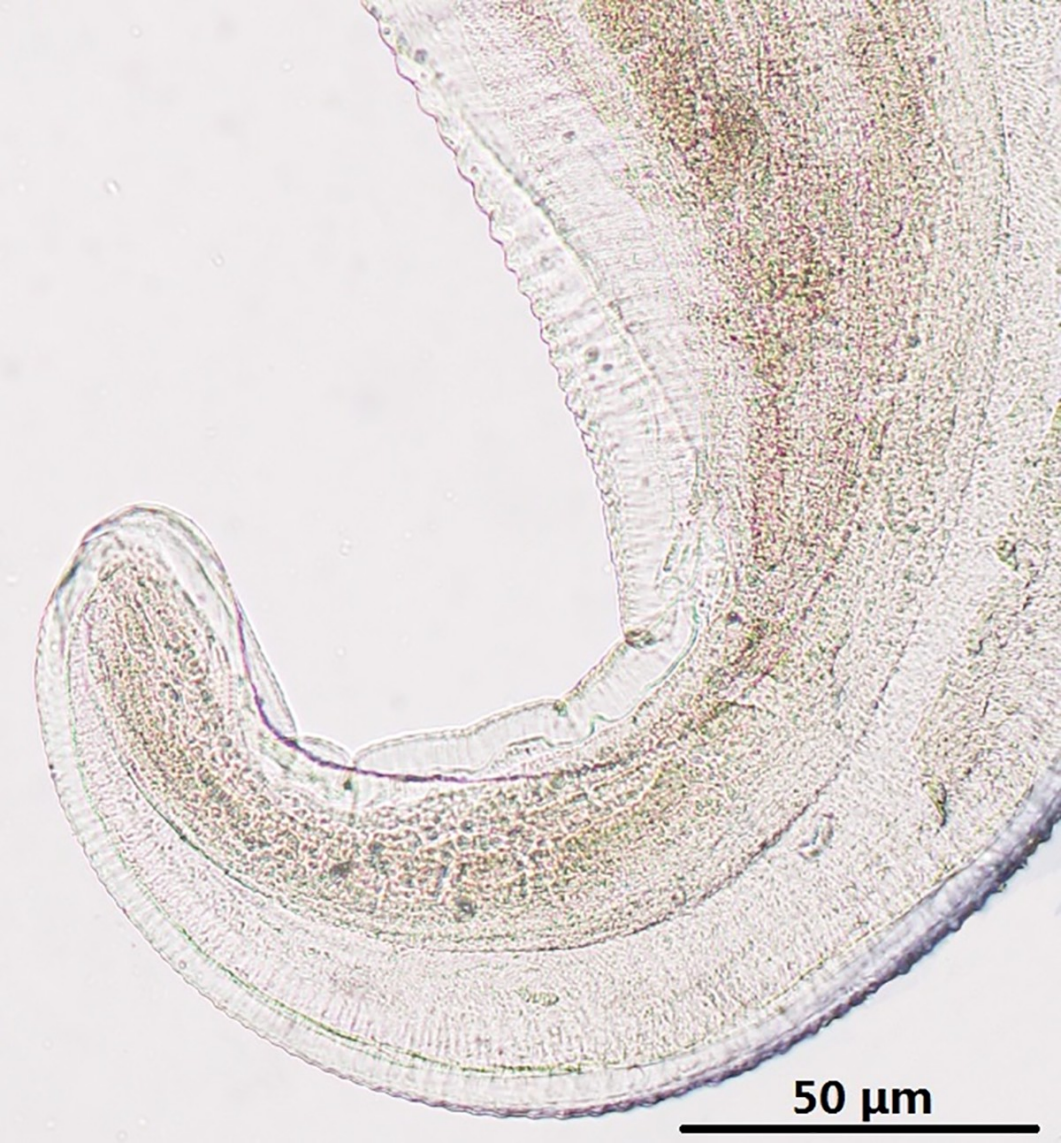
*Onchocerca fasciata*, female, light microscopy. Tail, lateral view.

**Fig 2:**
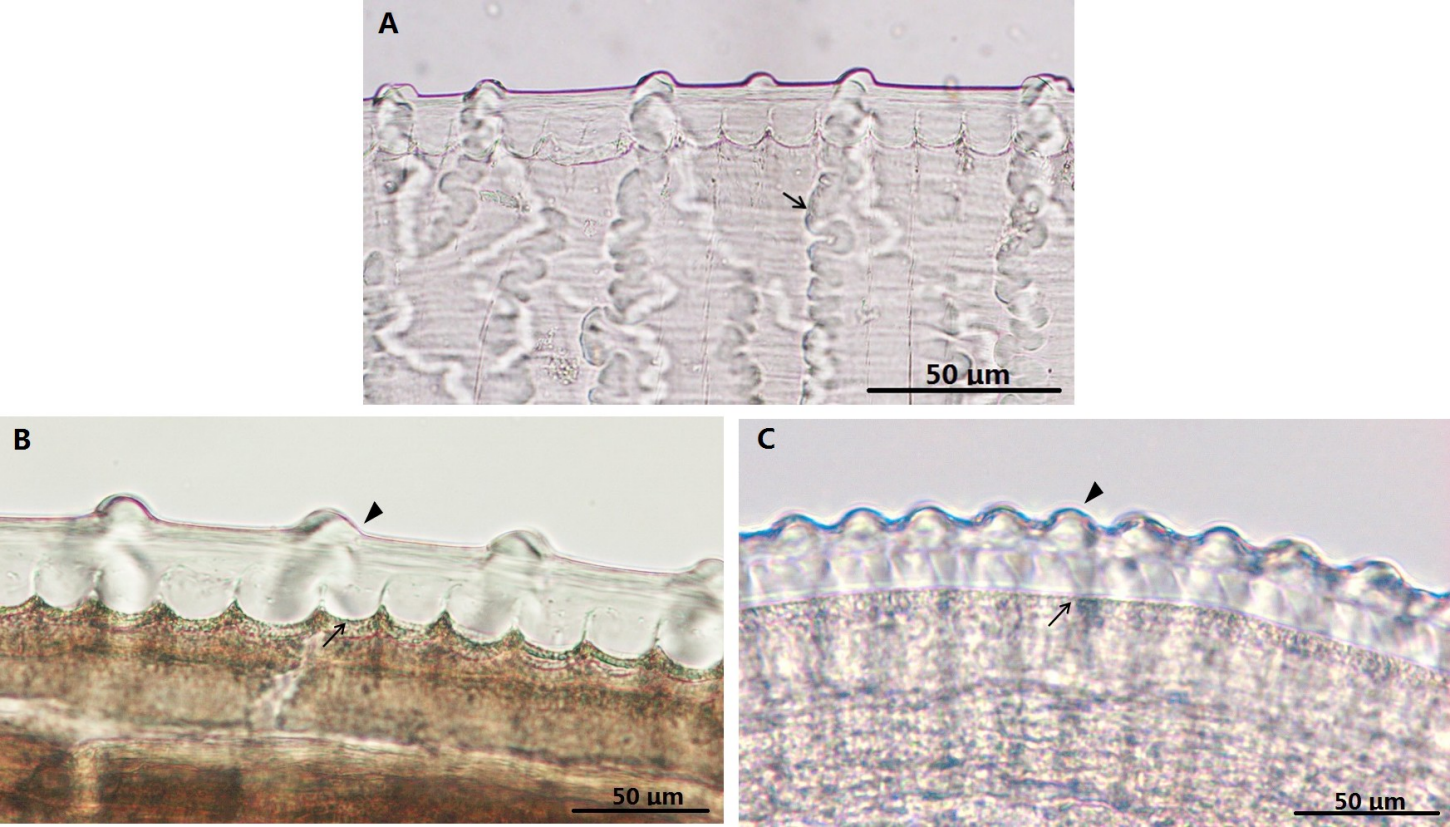
*Onchocerca fasciata*, female, light microscopy. A-C. Variations in the structure of the cuticle along the body. A. The ornament (arrow) at the mid-body. B. The relationship between ridges (arrowheads) to striae (arrows) at the mid-body. C. Region of the anterior body end showing the beginning of ridges and striae.

Females (based on two complete specimens, two anterior sections, fifteen posterior ends, and numerous portions of the mid-body): the average body length was 971.20 mm, and the width was 327–615 (386) μm. The total length of the oesophagus was 1.82–1.99 (1.92) mm with a muscular part length of 236–243 (240) μm, and the width at the level of the oesophago-intestinal junction was 65–90 (75) μm. The vulva was located 285–352 (327) μm from the anterior end. The diameter of the body at the level of the vulva was 180–111 (109) μm. The length of the tail was 163–201 (181) μm, with a pair of papilla located on a small tip (Fig 1). Irregular longitudinal striations were present on the outer surface, imparting a rough appearance (Fig 2A). The cuticle is composed of two distinct layers covering the body; the outer layer displayed transverse ridges that covered most of the body, often interrupting and branching on the lateral sides; the inner layer of cuticle is composed of bands called striae, of which there were usually four, rarely more, per ridge (i.e., one under the ridge and three between adjacent ridges). The ridges were small and close to each other and were 1.08–1.25 (1.15) mm away from the anterior end. The ridges became taller and farther apart in the posterior region, which was characterized by rounded ridges that were separated by 26–44 μm. In the mid-body fragments, the ridges were separated by spaces of up to 69 μm. Striae gradually disappeared near the ends of the body and were most prominent near the mid-body (Fig 2B), whereas striae on the anterior body end were 0.6 μm wide. Striae were larger (up to 1.2 μm wide) in the mid-body. The cuticle varied considerably in thickness and was thinnest at the anterior end (0.9–12.5 μm, gradually thickening to 31–38 μm). Ridges and striae were distinct, measuring 1.28–1.45 (1.31) mm from the posterior end. Where the ridges were 44–56 μm apart from each other, the striae were 13–19 μm wide and the cuticle was 25–38 μm thick. At approximately 87.5–110 (98.3) μm away from the posterior, the ridges were separated from each other by an 18–32 μm space, the striae were 7.5–10 μm wide, and the cuticle was 13–21 μm thick (Fig 2C). Near the tail, the ridges were close together and gradually faded. The striae decreased in size (0.3–0.8 μm wide), and the cuticle measured 0.3–0.7 μm.

**Fig 3:**
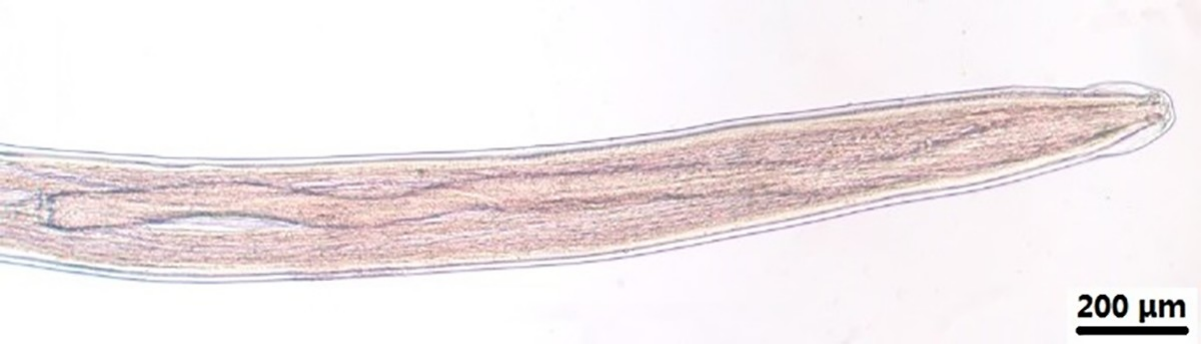
*Onchocerca fasciata*, male, light microscopy. Anterior end of the body, lateral view, showing the oesophagus.

**Fig 4:**
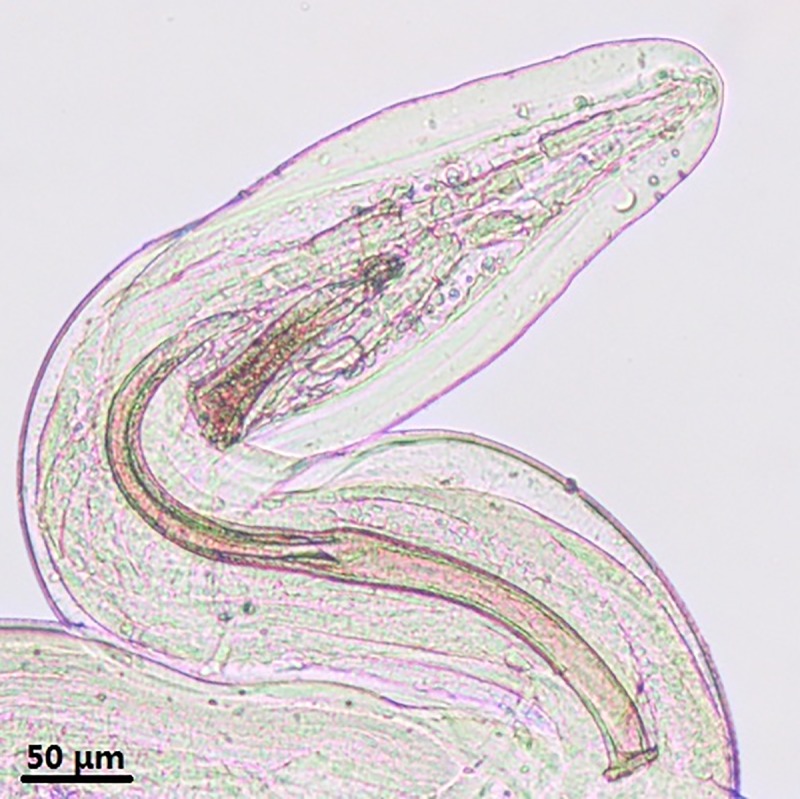
*Onchocerca fasciata*, male, light microscopy. Tail, ventral view, showing the spicules.

**Fig 5:**
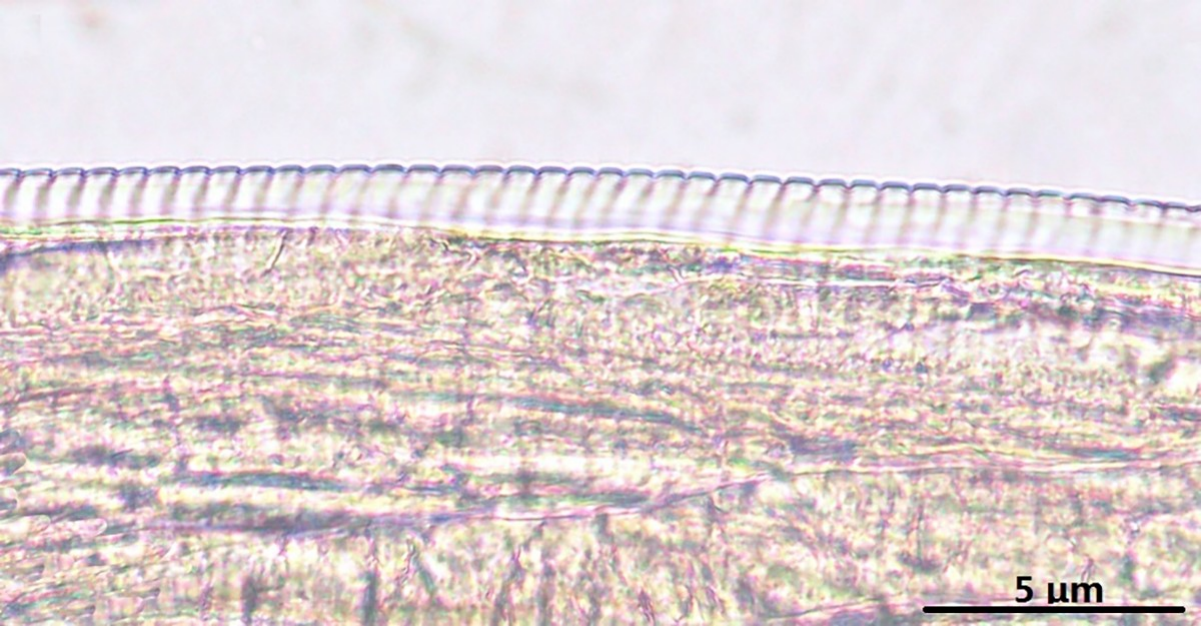
*Onchocerca fasciata*, male, light microscopy. Lateral view of the cuticle at the mid-body.

**Fig 6:**
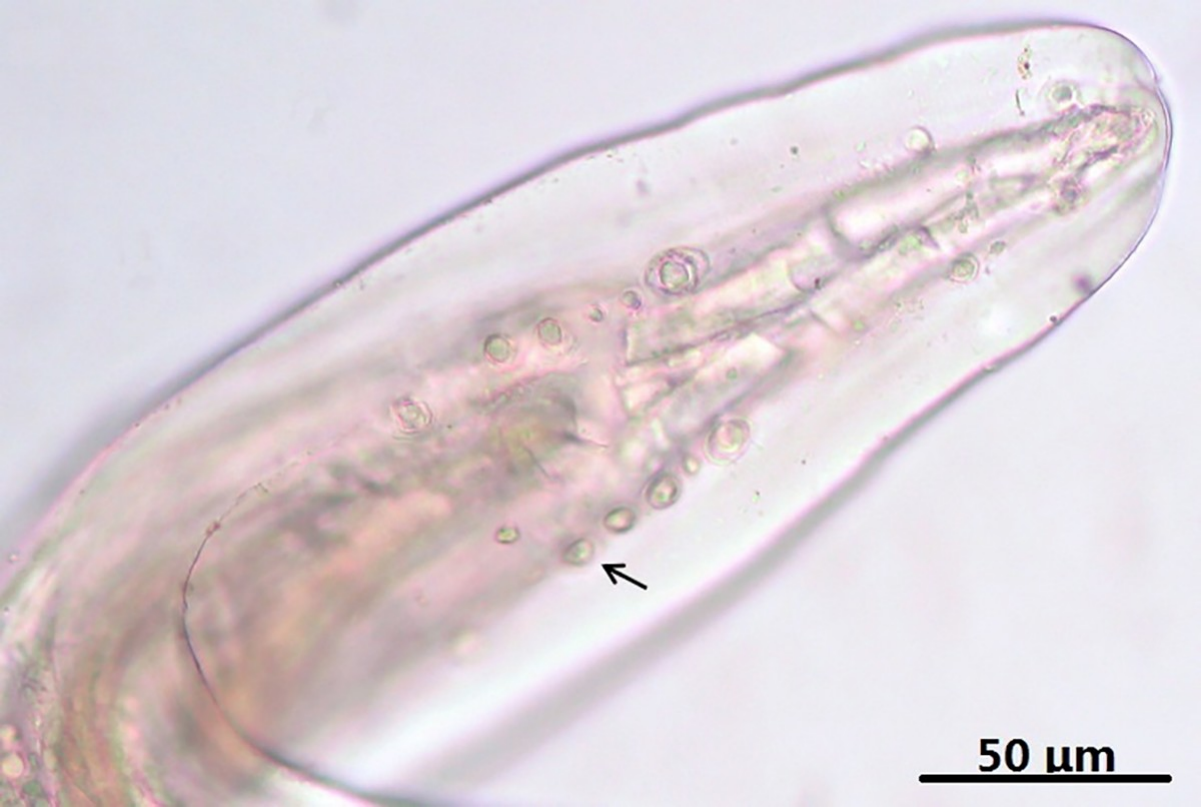
*Onchocerca fasciata*, male, light microscopy. Tail, ventral view, caudal papillae; note the second pair (arrow).

Males (based on five complete specimens, two anterior ends and one posterior end): male worms were smaller than females and were covered with a thin cuticle. Male worms were 63.00–88.00 (74.17) mm in length and 182–230 (202) μm wide. The nerve ring was located at 174–202 (194) μm from the anterior end. The total length of the oesophagus was 1.68–1.88 (1.75) mm and the width was 120–350 (202) μm (Fig 3). The tail was 125–188 (162) μm long and had a distinctive corkscrew-shaped end (Fig 4). The cuticle was comparatively thinner and simpler than that of females. The cuticle width was 7–9 (8) μm, and the length of the cuticular striation was 0.6 μm (Fig 5). Caudal alae were not obvious. Eight to nine pairs of caudal papillae were arranged in two groups. Near the cloacal aperture, there were two large precloacal pairs (two and three, respectively), two sub-ventral small pairs and one lateral large post-cloacal pair (likely pairs four, five, and six); the number and position of papillae varied. In the posterior tail region, there were three aligned pairs (seven to nine), and phasmids were located near the papillae of pair nine (Fig 6). At the end of the tail, there were two large fleshy structures that resembled papillae. The left spicule was 269–402 (319) μm long, tubular and tapered, stout and heavily cuticularized. It was divided into a proximal section 136–206 (167) μm in length and a distal portion 131–196 (155) μm in length and blade shaped with a bevelled end. The right spicule was 89–109 (100) μm long, broad, tubular, heavily proximally cuticularized, and narrowed to a knobbed distal end (Fig 4).

### Molecular analyses

**Fig 7:**
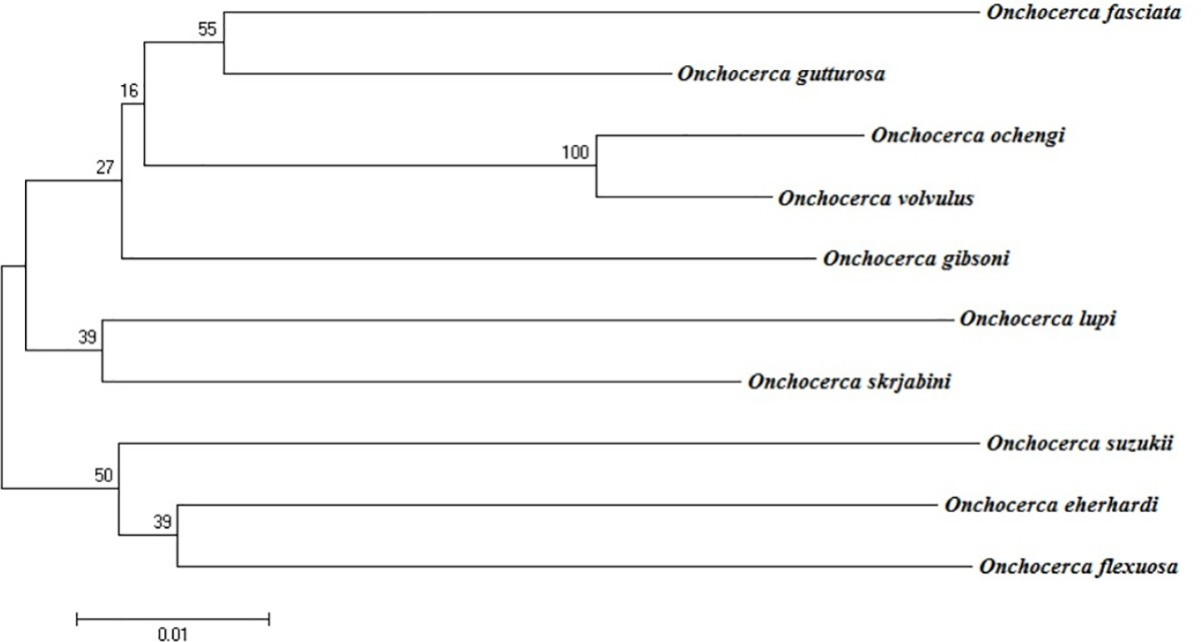
Phylogenetic tree of 10 Onchocerca species based on partial COI gene sequences constructed using the NJ method.

Sequences were compared to the *O*. *fasciata* sequence (JQ316672.1). Nucleotide differences in the COI gene among 10 species of *Onchocerca* are shown in Table 3. Phylogenetic trees were constructed using the NJ method (Fig 7). As shown in Table 3, the p-distances of the COI sequences were 0.02–0.11 for inter-species of *Onchocerca* and 0.06–0.10 in our sample of *O*. *fasciata* and other *Onchocerca* species.

**Table 3:** The number of nucleotide differences per site (p-distance) between COI sequences among *Onchocerca* species.

	1	2	3	4	5	6	7	8	9
**1. *Onchocerca fasciata***									
**2. *Onchocerca eherhardi***	0.10								
**3. *Onchocerca flexuosa***	0.10	0.08							
**4. *Onchocerca gibsoni***	0.08	0.10	0.10						
**5. *Onchocerca gutturosa***	0.06	0.09	0.09	0.06					
**6. *Onchocerca lupi***	0.10	0.11	0.10	0.09	0.08				
**7. *Onchocerca ochengi***	0.07	0.08	0.09	0.07	0.07	0.10			
**8. *Onchocerca skrjabini***	0.09	0.08	0.08	0.07	0.07	0.08	0.09		
**9. *Onchocerca suzukii***	0.10	0.09	0.09	0.10	0.08	0.11	0.09	0.09	
**10. *Onchocerca volvulus***	0.08	0.07	0.09	0.07	0.06	0.09	0.02	0.09	0.08

## Discussion

Camel Onchocerca nodules were described for the first time in 1909 in two animals imported from Karachi to Australia and were first described in Indian dromedary camels by Railliet and Henry (1910) [14]. Studies investigating the different stages of camel onchocercosis, histopathology and the characterization of ultrastructures were conducted by Nasher AK (1986), Ghandour *et al*. (1991), Determann *et al*. (1997), Aida A *et al*. (2000), N.Moghaddar *et al*. (2006) and Khodakaram-Tafti *et al*. (2010) [18,19,22–25]. However, previous reports of camel onchocercosis focused on *Camelus dromedarius*, which is only one species of camel. In this study, we examined *O*. *fasciata* that parasitized *C*. *bactrianus*.

The reported prevalence of *O*. *fasciata* varies considerably and has been determined to be as low as 2.75% in Sudan and Somalia [22] and as high as 59.0% in Saudi Arabia [23], although other studies found intermediate prevalence Sudan and Somalia (15.5%) [15], Saudi Arabia (34.2%) [15] and (33.3%) [18]; Iran (48%) [24] and (6.0%) [25]. We investigated the disease prevalence in two-humped camels and detected an infection rate of 80.3% in camels sampled from Inner Mongolia. In Alashan and Bayannaoer, the infection rates were 88.9% and 51.4%, respectively. High variation was observed in the infection rates of different species and countries. This variation could be attributed to exposure to insect vectors and host interspecific differences. In our study, increased camel age was correlated with the degree of calcification, which was similar to the results reported by Ghandour (1991) [18].

Clinical lesions attributed to *O*. *fasciata* were commonly confined to the subcutaneous connective tissues of the neck and other parts of the body, such as the elbow and the abdomen. The lesions were associated with pain, swelling, ulcerations, and the presence of female nematodes [18,22,25]. However, we found that fibrous tissue nodules were only recovered from the necks of camels. Therefore, the incidence of onchocercosis and the distribution of local vectors were deduced based on lesion pathology. Blood-sucking insects, particularly *Simuliidae* and *Culicoides*, are known vectors of *Onchocerca* [26,27]. In contrast, Crosskey and Buttiker (1982) [28] speculated that *Simuliidae* sp. were unlikely vectors of *O*. *fasciata* in Saudi Arabia. In contrast, Muller (1979) [29] believed that *Culicoides* spp. were far more prevalent and were more likely to be vectors. In this study, we also found blood-sucking insects, such as midges and mosquitos, in the river close to where the camels lived. Further research is necessary to determine the vectors of *Onchocerca*.

Early studies were based on the use of a few worm fragments to describe the morphological characteristics of the nematodes in camels, and the data were separately reported in faunistic, epidemiological, or histopathological studies. *O*. *fasciata* was initially described by Railliet and Henry (1910) [14] and subsequently re-described by Henery and Masson (1933) [30]. These studies described the body dimensions, structure of the female cuticle, length of the male spicules and oesophagus length in females (1.82–1.99 vs. 1 mm). Although there were differences, they were within the limits of the known intraspecific variation for this species. A complete comparison of our results with previous results was difficult due to the lack of morphological studies for this parasite. The present re-description of *O*. *fasciata*, based on samples collected from Bactrian camels, revealed many details that were described and suitably illustrated and provided important morphological data on the spicules and the cuticle structure of the worms. We described the tail of the male in detail, which was easily observed and prominent.

The cuticle of each gender showed considerable variation in thickness and structure. In males, the cuticle was thinner and had a simple striation pattern. However, in females, the cuticle was thick and more prominently ornamented. The cuticle structures in nematodes are important morphological features for species identification in *Onchocerca*. Similar morphological characteristics of the cuticular ridges and striation have been reported in several other species, such as *O*. *gibsoni* (Chodnik, 1957), *O*. *armillata*, *O*. *gutturosa* (Chauhan & Pande, 1978), *O*. *lienali*, *O*. *stilesi* (Eberhard, 1979) and *O*. *lupi* (Demiaszkiewicz and Matsaberidze, 1991) [31–34].

The nematodes found in Inner Mongolian camels were further identified as *O*. *fasciata* using molecular biology methods. Phylogenetic analysis of the COI genes of the 10 *Onchocerca* species indicated that there were two obvious branches. However, *O*. *flexuosa* was not a primitive species and differed from the description of Krueger (2007) [17]. This discrepancy may be attributable to the genes that were selected for analysis and the different parasite strains. Phylogenetic tree analysis revealed distant relationships between *O*. *skrjabini* and *O*. *eherhardi* isolated from Japanese deer. Similarly, *O*. *gibsoni*, *O*. *gutturosa* and *O*. *ochengi* were separate from cattle and were also distantly related. However, *O*. *fasciata* isolated from camels was the closest relative to *O*. *gutturosa*, which was isolated from cattle. Based on these results, species with different hosts may be closely related to each other. Molecular biology methods are widely used in the classification of *Onchocerca* to validate the results of morphological methods. This technology is used to analyse potential hosts and vectors for similar *Onchocerca* species.

O. *fasciata* has a widespread geographical distribution. In addition to infecting the single-humped camels found in Australia, Saudi Arabia, Egypt, Iran, Sudan and Somalia [18,22,24,25], this species parasitizes the two-humped camels of Inner Mongolia, China. These data reveal that its vector is widely distributed around the world.

## Conclusions

In this study, we determined *O*. *fasciata* infection rates and nodule distribution in camels from Inner Mongolia. The morphology of *O*. *fasciata* described herein provides a better understanding of and reliable documentation for the characterization of other *Onchocerca* species. Our results will provide insight into the pathogenicity of *O*. *fasciata* in *C*. *bactrianus* from Inner Mongolia, China. Further studies investigating the vectors of *O*. *fasciata* are being conducted in our lab with the aim of controlling onchocerciasis.

## Supporting information

S1 Fig The female of *Onchocerca fasciata* for light microscopy. A. Tail, lateral view. B1-3. Variations in the structure of the cuticle along the body. B1. The ornament (arrow) at the mid-body. B2. Region of the anterior body end, showing the beginning of ridges and striae. B3. The relationship between ridges (arrowheads) to striae (arrows) at the mid-body.
(TIF)

S2 Fig The male of *Onchocerca fasciata* for light microscopy. A. Anterior end of the body, lateral view, showing the oesophagus. B. Tail, ventral view, showing the spicules. C. Lateral view of the cuticle at the mid-body. D. Tail, ventral view, caudal papillae; note the second pair (arrow).
(TIF)
