# Supplementary figures and images for: A survey of Onchocerca fasciata infection in camels (Camelus bactrianus) with notes on morphology
Source: PLoS One. 2019 Apr 4;14(4):e0214477. doi: 10.1371/journal.pone.0214477 (PMC6448845; doi:10.1371/journal.pone.0214477)

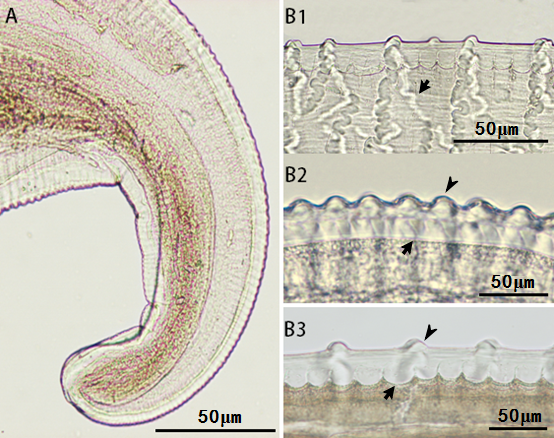

Supplement: S1 Fig — A. Tail, lateral view. B1-3. Variations in the structure of the cuticle along the body. B1. The ornament (arrow) at the mid-body. B2. Region of the anterior body end, showing the beginning of ridges and striae. B3. The relationship between ridges (arrowheads) to striae (arrows) at the mid-body. (TIF) [file pone.0214477.s001.tif]

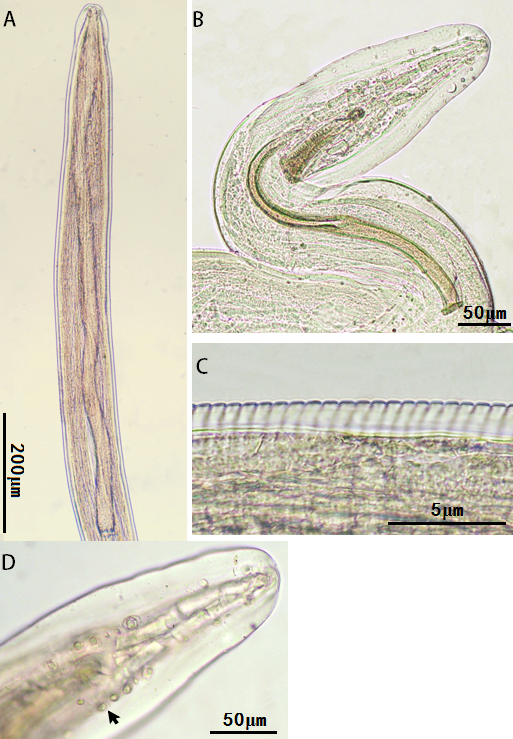

Supplement: S2 Fig — A. Anterior end of the body, lateral view, showing the oesophagus. B. Tail, ventral view, showing the spicules. C. Lateral view of the cuticle at the mid-body. D. Tail, ventral view, caudal papillae; note the second pair (arrow). (TIF) [file pone.0214477.s002.tif]
